# Incorporation of the Trifluoromethylthio Group (CF_3_S−) into Biomolecules: A Versatile Tool for Advancing Life Sciences

**DOI:** 10.1021/acs.joc.6c00904

**Published:** 2026-06-12

**Authors:** Jure Gregorc, Nathalie Lensen, Grégory Chaume, Jernej Iskra, Thierry Brigaud

**Affiliations:** † University of Ljubljana, Faculty of Chemistry and Chemical Technology, Večna pot 113, 1000 Ljubljana, Slovenia; ‡ 27004 CY Cergy Paris Université, CNRS, BioCIS, 95000 Cergy Pontoise, France; § Université Paris-Saclay, CNRS, BioCIS, 91400 Orsay, France

## Abstract

The trifluoromethylthio functional group (CF_3_S−) has gained considerable attention in synthetic and medicinal chemistry due to its ability to enhance the pharmacokinetic profiles of functionalized compounds. This Synopsis highlights recent progress in the incorporation of the CF_3_S group into biomolecules and bioactive compounds, with a focus on its influence on physicochemical properties and its applications in medicinal chemistry and chemical biology.

## Graphical abstract

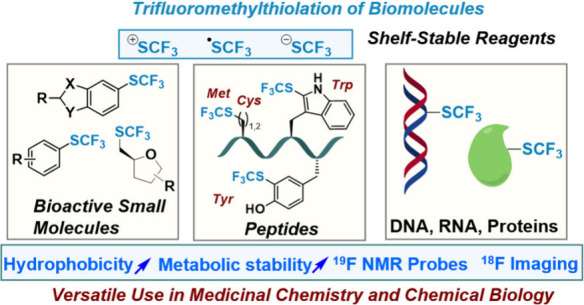

The association of fluoroalkyl groups with chalcogen atoms leads to functional groups with increased lipophilicity and tunable electronic properties, often significantly affecting the acidity or basicity of proximal moieties.^1^ While such characteristics are highly desirable in drug optimization programs, chalcogen-associated substituents (R–XR_F_) remain underrepresented in FDA-approved pharmaceuticals compared to monofluorine (R–F) and trifluoromethyl (R–CF_3_) chemotypes.^2^ Among these emerging motifs, the trifluoromethylthio group (CF_3_S−) is of particular interest due to its high lipophilicity and strong electron-withdrawing character.^3,4^ Compared to CF_3_, the CF_3_S group is significantly more lipophilic (π = 1.44) and exhibits comparable electronic effects. Moreover, the CF_3_S group features three magnetically equivalent fluorine atoms in an isolated spin system, rendering it a sensitive ^19^F NMR probe for structural and dynamics studies of biomolecules such as peptides,^5,6^ proteins,^7–10^ and nucleic acids.^11–15^

Over the past 15 years, numerous shelf-stable reagents for direct trifluoromethylthiolation of structurally complex substrates have been developed.^4,16,17^ These advances have been complemented by indirect strategies involving trifluoromethyl­ation of sulfur-containing precursors, such as thiols or disulfides.^18^ Collectively, the methodology for CF_3_S introduction has undergone substantial development and has been comprehensively reviewed elsewhere.^4,16,19,20^ In addition to these reviews dedicated to synthetic aspects, this Synopsis provides a focused overview of recent advances in the incorporation of the trifluoromethylthio group into bioactive small molecules and biomolecules, including amino acids, peptides, and oligonucleotides, emphasizing the pronounced effect of trifluoromethylthiolation on their physicochemical and pharmacokinetic properties. We summarize the use of CF_3_S– substitution in medicinal chemistry, particularly in structure–activity relationship (SAR) studies, and highlight its potential use in chemical biology as a probe for ^19^F NMR spectroscopy and ^18^F-PET imaging.

## 1 CF_3_S-Containing Bioactive Small Molecules

Rational incorporation of lipophilic groups can enhance membrane permeability and metabolic stability, therefore, trifluoromethylthiolation is regarded as a promising strategy for optimizing the pharmacokinetic properties of biomolecules and bioactive compounds.^21^ Representative examples of trifluoro­methylthiolated drug candidates in clinical use or advanced trials, together with CF_3_S-containing biologically active compounds are shown in Figure 1.

**Figure 1:**
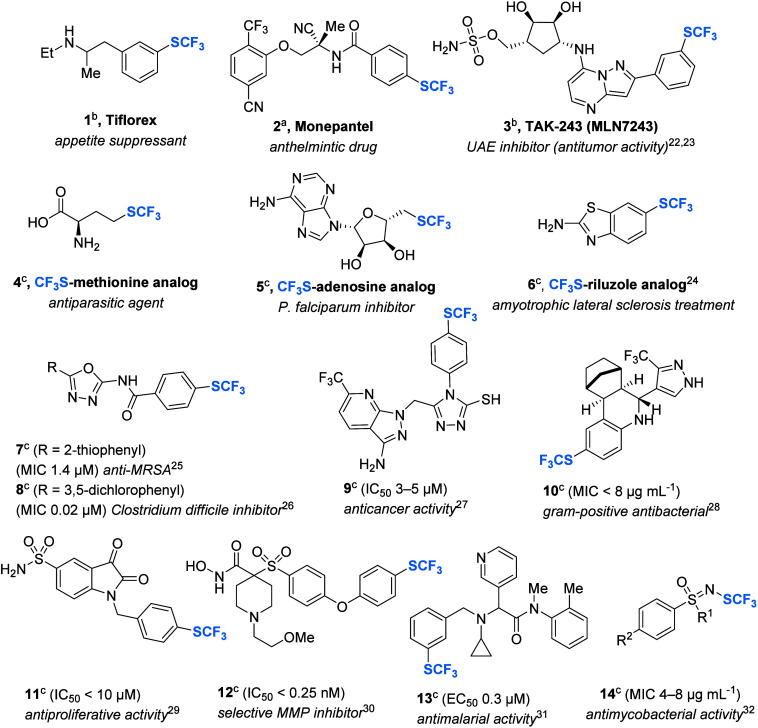
Selected CF_3_S-containing registered pharmaceutics,^a^ those in clinical trials,^b^ and biologically active compounds.^c^ ^22–32^

The development of the potent antitumor agent TAK-243 (**3**, formerly MLN7243) exemplifies a recent exploitation of CF_3_S group properties.^22^ TAK-243 has been proposed to adopt a planar conformation that enables strong anchoring within the hydrophobic binding pocket, while the *meta*-CF_3_S substituent likely acts as a hydrophobic hook, enhancing binding through favorable contacts between the trifluoro­methyl­thiobenzene moiety and the protein’s hydrophobic patch.^23^

An inspiration for the design of new biologically active compounds was the synthesis of SCF_3_ analogues of known drugs containing the OCF_3_ group. A study on ADME properties of the SCF_3_ analogue of the riluzole drug **6** (Figure 1) further highlighted the non-metabolizable lipophilicity effect of the trifluoromethylthio group and its potential to prolong *in vivo* half-life.^24^ Replacement of oxygen by sulfur led to an increase in lipophilicity of 0.5 log*P* and a reduced solubility, which was attributed to higher lattice energy. The CF_3_S-analogs retained stability in human liver microsome and showed no inhibition of CYP3A4. *In vitro* safety profiling revealed no significant off-target activity, indicating that the CF_3_S group does not present intrinsic safety concerns.

The trifluoromethylthio group’s ability to enhance *in vitro* biological activity has been explored in multiple SAR studies. Selected successful examples of potent biological active compounds that emerged from these studies are highlighted in Figure 1. This literature survey suggests that the trifluoromethylthio group is typically introduced via convergent strategy, using robust couplings of commercially available CF_3_S-substituted benzene derivatives. In general, trifluoromethylthiolated bioactive analogs demonstrated increased metabolic stability, improved bioavailability and low toxicity in human cell lines at pharmacologically relevant concentrations. An exception is a class of antibacterial *N*-trifluoromethylthiosulfonimidamides and sulfoximines **14**,^32^ which exhibited high cytotoxicity in human cell lines, a property not observed for the *C*-SCF_3_ compounds **1**–**13** (Figure 1). SAR analysis attributed the cytotoxicity to the *N*-SCF_3_ group, as the corresponding *N*-CF_3_ analogs displayed neither antibacterial nor cytotoxic activity.^32^ The N–SCF_3_ bond is presumed to be more labile than the C–SCF_3_ bond and could release the trifluoromethanethiol toxicophore. This potential liability should be considered in future applications of the trifluoromethylthio group in drug design.

## 2 Trifluoromethylthiolated Amino Acids and Peptides

### Trifluoromethionine and Trifluoromethylcysteine Derivatives

Until recently, the synthesis and incorporation of trifluoro­methylcysteine (TfmCys) and trifluoromethionine (TFM) into peptides have dominated reports on CF_3_S-containing amino acids (AAs).^33,34^ The main strategies for accessing TfmCys and TFM building blocks include radical trifluoro­methylation of (homo)­cystines using CF_3_SO_2_Na/*t*-BuOOH or electrophilic trifluoromethylation of protected (homo)­cysteines using the Togni hypervalent iodine­(III)–CF_3_ reagent (Scheme 1).^35,36^ More recently, TfmCys synthesis was reported within a broad scope of substrates undergoing efficient electrophilic *S*-trifluoromethylation using the CF_3_-thianthrenium triflate reagent.^37^ Moreover, nucleophilic trifluoromethylthiolation of 1,2- and 1,3-sulfamidates with [Me_4_N]­SCF_3_ enabled the preparation of optically pure fluorinated derivatives of cysteine, homocysteine and β-methylated cysteine in 58–98% yield.^38^

**Scheme 1:**
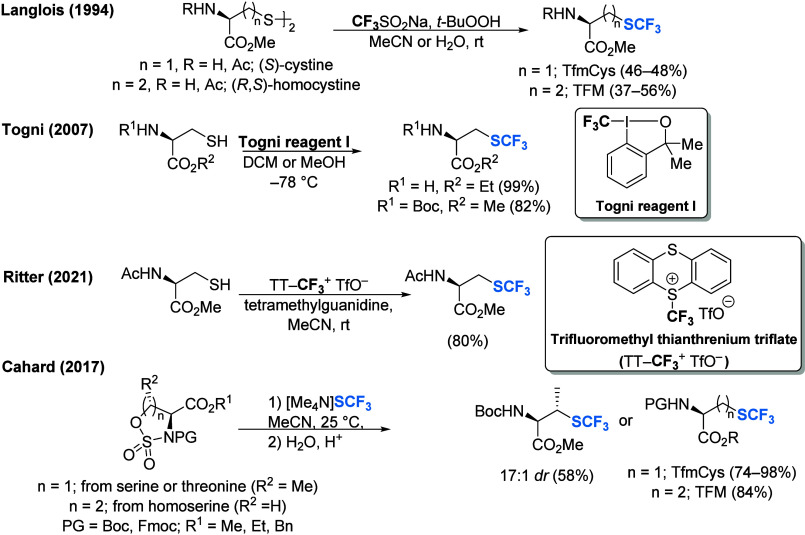
Reported Synthetic Routes toward TFM and TfmCys Derivatives

The incorporation of TFM and TfmCys into peptides has mainly been achieved in solution *via* standard peptide coupling^39^ or by late-stage *S*-trifluoromethylation of Cys-containing peptides.^40^ The latter was demonstrated by Togni, Seebach and co-workers using their hypervalent iodine­(III) trifluoromethylation reagent (Scheme 2). The reaction yielded TfmCys-containing dipeptides in 55–92% yields with fairly good functional group tolerance. However, C2-trifluoro­methyl­ation of the tryptophan residue of Octreotide was observed as a side reaction.^40^ The authors also noted the CF_3_S group β-elimination from TfmCys in basic media, implying its inherent limitations relative to TFM in peptide chemistry applications.^40^

**Scheme 2:**
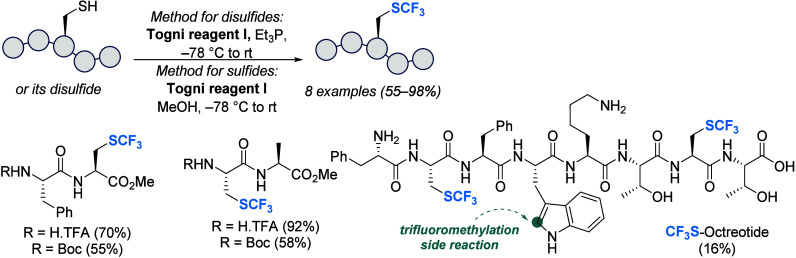
Direct *S*-Trifluoromethylation of Cys Residues in Selected Peptides Using the Togni Reagent^40^

The Noël group later developed a visible-light photocatalytic method for *S*-trifluoromethylation of Cys and Cys-containing dipeptides using CF_3_I in both batch and in continuous flow.^41^ Following a single electron transfer event, the transient electrophilic trifluoromethyl radical reacts rapidly to form the S–CF_3_ bond, affording TfmCys derivatives in moderate to excellent yield while avoiding disulfide byproduct formation.

The incorporation of TFM and particularly TfmCys into peptides *via* solid-phase peptide synthesis (SPPS) has been scarcely reported until recently.^6,42–44^ Our group has reported both solution-phase and solid-phase strategies for incorporating TfmCys and TFM to probe the hydrophobic effects of CF_3_S in peptides.^45,46^ Boc/Bn-protected building blocks were prepared by radical trifluoromethylation in moderate yield (30–42%) and incorporated into tripeptides by iterative solution phase peptide coupling (Scheme 3).^45^ *N*-Boc-protected TfmCys was also incorporated by standard Fmoc-SPPS in comparable yield (30%). Furthermore, late-stage *S*-trifluoromethylation of disulfide-linked tripeptides using the iodine­(III)–CF_3_ reagent was also demonstrated (Scheme 3).^45^

**Scheme 3:**
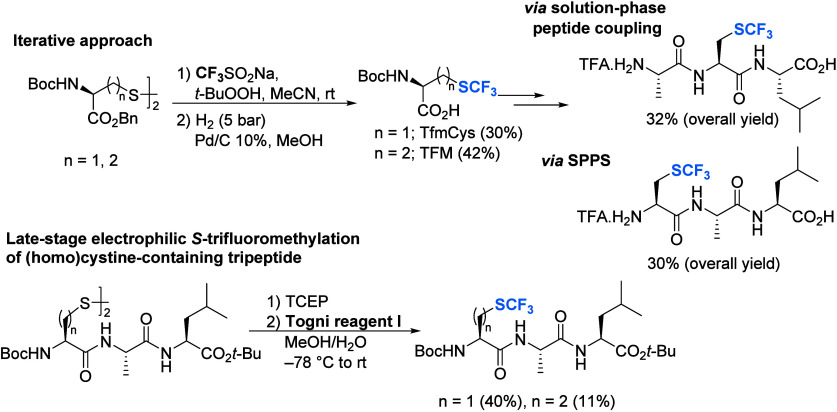
Solution-Phase or Solid-Phase Integration of TfmCys and TFM in Tripeptides and Late-Stage Trifluoromethylation of (Homo)­cystine-Containing Peptides Reported by Our Group^45^

Furthermore, in our studies on fluorinated peptide-based hydrogels, we evaluated the importance of aromaticity versus hydrophobicity by introducing TFM, among other fluorinated amino acids (FAAs), in a hexapeptide hydrogel sequence.^43^ Fmoc-TFM was prepared on gram-scale in 34% overall yield and excellent purity and subsequently used in standard SPPS (Scheme 4A). Replacement of a phenylalanine residue in the reference peptide hydrogel (**P1**) with TFM at the *C*-terminus (**P19**) afforded a hydrogel peptide with similar CD profile to **P1** but with increased storage (G′) relative to the elastic properties of the material and loss moduli (G″) describing the viscous portion of the material. Although **P19** formed more polymorphic fibers, it exhibited good *in vitro* release properties and a comparable *in vivo* stability profile to the reference peptide **P1**.

**Scheme 4:**
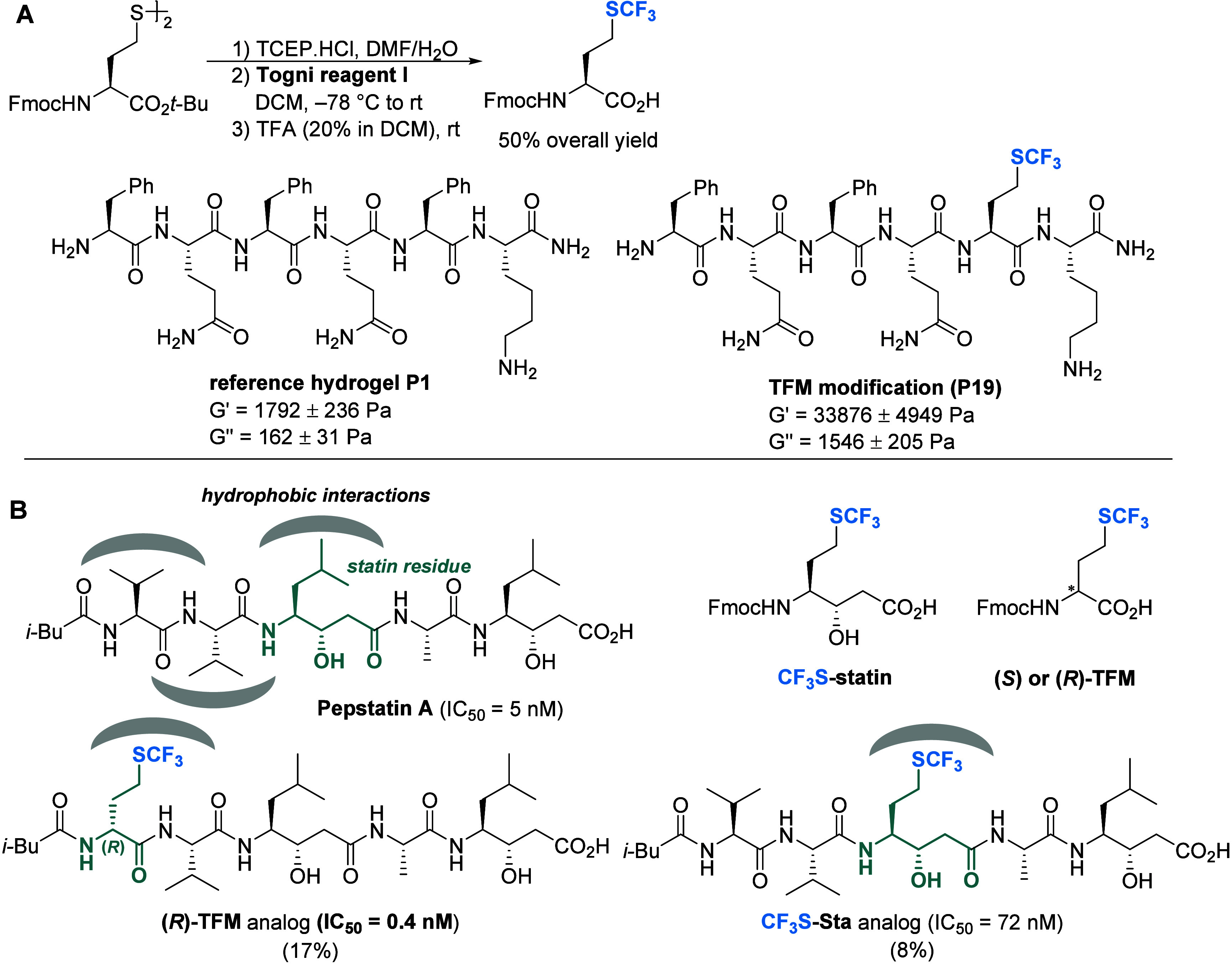
(A) Gram-Scale Synthesis of TFM Building Block and Representative Hexapeptide Hydrogels;^43^ (B) Pepstatin A and Pepstatin A-Based CF_3_S-Peptides with Nanomolar Cathepsin D Inhibitory Activity^44^

In 2024, Pytkowicz and co-workers reported the solid-phase synthesis and biological evaluation of trifluoromethylthiolated pepstatin A analogs as potent inhibitors of Cathepsin D (CD) (Scheme 4B).^44^ To improve the pharmacokinetic properties of pepstatin A, CF_3_S-Statin and (*S*)- or (*R*)-TFM residues were incorporated into the sequence. The ligand where the first valine was replaced by (*R*)-TFM displayed sub-nanomolar IC_50_ against CD. This increased potency is likely due to enhanced hydrophobic interactions with the lipophilic residues in the catalytic pocket, as supported by molecular docking study.

### Trifluoromethylthiolated Aromatic Amino Acid Derivatives

In 2012, Billard and co-workers first demonstrated the reactivity of tryptophan, among other C3-substituted indoles, toward direct electrophilic trifluoromethylthiolation using their first-generation trifluoromethanesulfenamide reagent (Scheme 5A).^47^ While this method was effective for tryptamine and other C3-substituted indoles, tryptophan analogs showed low conversion (13–16%) and were not successfully isolated. Nevertheless, this work demonstrated the feasibility of direct CF_3_S-incorporation into activated aromatic AA residues and prompted further investigation. In 2023, our group made progress in the synthesis of CF_3_S-Trp derivatives, achieving quantitative conversion of tryptophan derivatives to C2-SCF_3_ analogs using a more effective *para*-chloro trifluoromethanesulfenamide reagent in combination with either trifluoro­methane­sulfonic acid or BF_3_·OEt_2_ as activators (Scheme 5B).^48^ Under optimized conditions, a series of C2-trifluoromethylthiolated tryptophan analogs with varied terminal protecting groups (PGs) were synthesized (>90%) together with CF_3_S-analogs of biologically important tryptamines (66–92%). The reaction scope was extended to tyrosine and DOPA derivatives, featuring less-activated phenolic or catechol moieties (Scheme 5C).^48^ In these cases, a slightly larger reagent excess was required to achieve quantitative conversion, affording products in moderate to high yield (68–97%). For DOPA or dopamine derivatives, the additional hydroxyl group’s directing effect changed the regioselectivity of CF_3_S-incorporation.

**Scheme 5:**
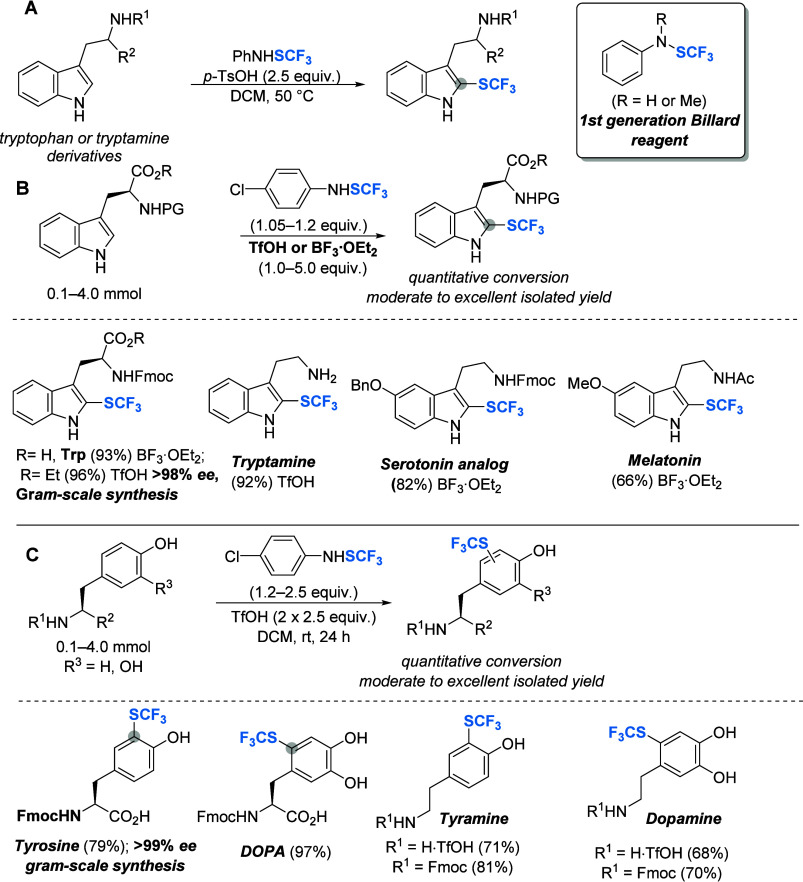
(A) Direct Electrophilic Trifluoromethylthiolation of Tryptophan Derivatives Using the First-Generation Trifluoromethanesulfenamide Reagent;^47^ Scope of Our Electrophilic Trifluoromethylthiolation Method for (B) Tryptophan and (C) Tyrosine/DOPA Analogs^48^

Recently, Sutherland and co-workers developed a Lewis acid/base dual catalytic system using *N*-trifluoromethylthiosaccharin, enabling trifluoromethylthiolation of less-activated arenes (Scheme 6A).^49^ This reaction is effective for a wide range of complex and biologically significant substrates including *N*-Cbz-protected tyrosine derivative, which was isolated in 70% yield as a single CF_3_S-regioisomer. Based on our literature review, no trifluoromethylthiolated histidine analogs have been reported so far while only one phenylalanine derivative has been synthesized from the corresponding aryl iodide.^50^ The reaction proceeds *via* photoredox-mediated trifluoromethylthiolation using a bench-stable Ni­(II) salt and an iridium photocatalyst in combination with AgSCF_3_ affording the CF_3_S-Phe analog in 62% yield (Scheme 6B).

**Scheme 6:**
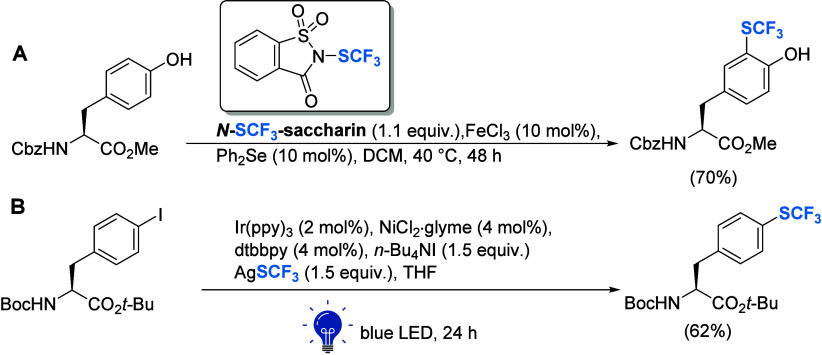
Other Strategies for the Synthesis of CF_3_S-Functionalized Aromatic Amino Acids: (A) Lewis Acid/Base Dual Catalysis;^49^ (B) Ni-Catalyzed Photoredox-Mediated Trifluoromethylthiolation^50^

Using our protocol, enantiopure SCF_3_-containing *N*-Fmoc-protected tryptophan, tyrosine and dimethyltyrosine (Dmt) building blocks were synthesized on gram-scale in good to excellent yield (66–93%; see Scheme 5). These aromatic SCF_3_-containing amino acids and trifluoromethionine (TFM) were used in standard Fmoc-SPPS to prepare a series of trifluoromethylthiolated opioid peptides analog to endomorphin-1 (EM1) (Scheme 7).^48,51^ *In vitro* μ-opioid receptor (μOR) binding assays demonstrated that these fluorinated analogs retained binding affinity and potency accompanied by an increased hydrophobicity. The (CF_3_S)­Dmt-containing analog showed the most favorable profile (*K*_*i*_ = 1.4 nM, EC_50_ = 0.9 nM) and revealed a significant increase in half-life plasma stability studies (72-fold relative to EM1).^51^

**Scheme 7:**
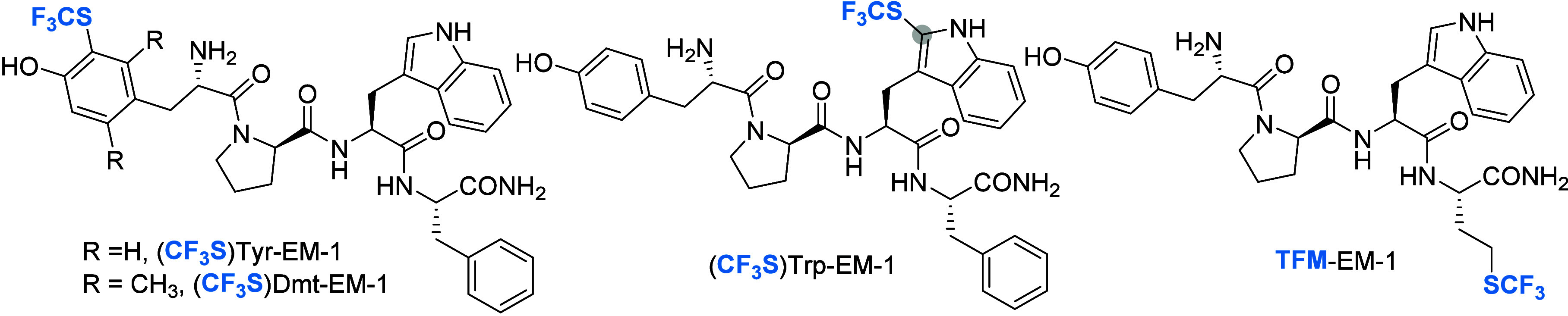
Synthesized Endomorphin-1 (**EM1**) Analogs Incorporating (CF_3_S)­Tyr, (CF_3_S)­Dmt, (CF_3_S)­Trp and TFM^48,51^

Complementary to SPPS, we developed late-stage C2-trifluoromethylthiolation of tryptophan residues in short peptides in moderate to high yield (66–80%, Scheme 8), demonstrating excellent Trp vs Tyr selectivity.^48^ The late-stage reaction was also successfully applied to **EM1** consisting of three aromatic AAs.

**Scheme 8:**
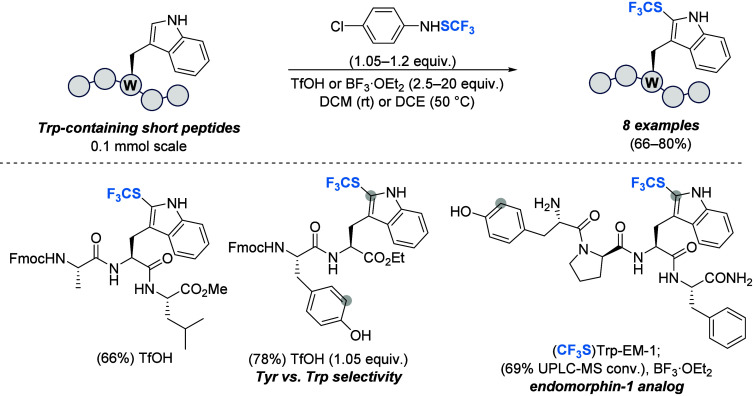
Late-Stage C2-Trifluoromethylthiolation of Trp Residues in Short Peptides and Endomorphin-1 ^48^

Recently, Li and co-workers reported tryptophan diversification in native peptides *via* late-stage catalyst-free electrophilic C2-sulfenylation (Scheme 9).^52^ The reaction employs quinoline-based thiosulfonates (QSO_2_SR_F_) as electrophilic SR_F_ sources (sulfur-associated fluoroalkyl groups) under trifluoroacetic acid (TFA) conditions and proceeds with high chemoselectivity, showing compatibility with other canonical AAs, disulfide linkages, and *O*-glycosylated peptides. The use of TFA as the solvent was crucial as it enhances the electrophilicity of the SR _F_ moiety *via* H-bonding interaction with the quinoline ring and has high peptide dissolving capability. An impressive library of model peptides and marketed peptide drugs was subjected to C2-trifluoromethyl­thiolation and isolated in exceptionally high yields relative to peptide post-modification protocols (35–93% after HPLC purification). In the case of the bioactive peptide melittin, the CF_3_S analog demonstrated increased stability in human serum (from 8 h to >24 h) while retaining anticancer activity comparable to that of native melittin.

**Scheme 9:**
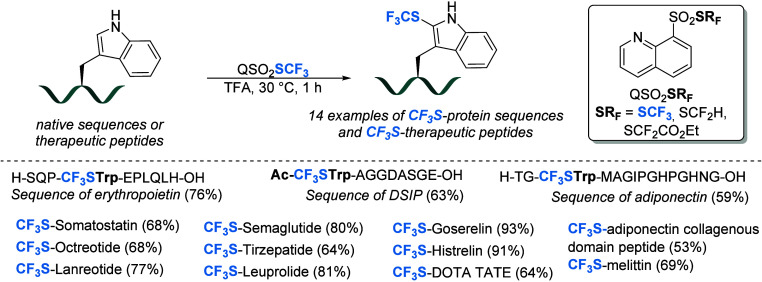
Late-Stage Electrophilic C2-Sulfenylation of Trp Residues Using Quinoline-Based Thiosulfonates and Selected Examples from Protein Sequences and Peptides Drugs^52^

### Miscellaneous Trifluoromethylthio-Containing Amino Acids

Thiols have been reported to undergo electrophilic *S*-trifluoromethylthiolation with trifluoromethanesulfenamides, yielding *S*-SCF_3_ disulfide analogs.^53^ As an example, *S*-trifluoromethylthiolated cysteine derivative was isolated in 58% yield (Scheme 10A).^53^ Furthermore, Zhang and co-workers developed a hypervalent trifluoromethylthio-iodine­(III) reagent (TFTI) that enabled efficient *S*-trifluoromethyl­thiolation of thiols, including cysteine and cysteine-containing dipeptides, under mild HFIP activation (70–88%, Scheme 10A).^54^ Very recently Li and co-workers also reported the late-stage penicillamine-selective *S*-sulfenylation in native peptides *via* thiosulfonate chemistry.^55^

**Scheme 10:**
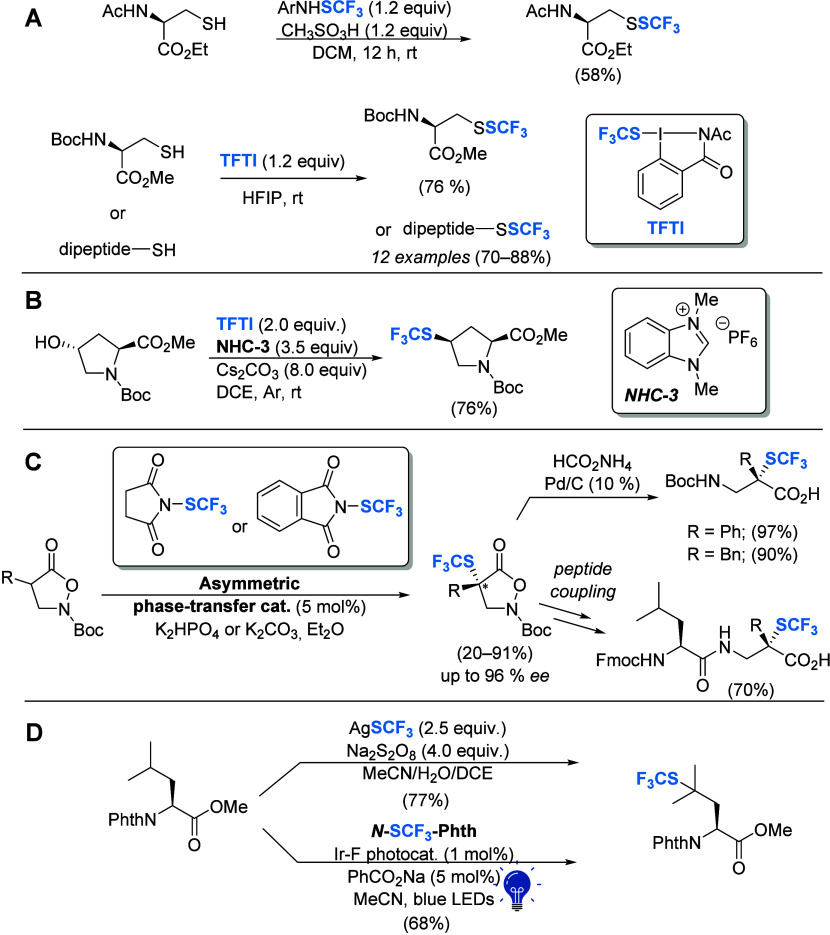
Examples of Miscellaneous CF_3_S-AA Syntheses: (A) *S*-SCF_3_ Analogs of Cys;^53,54^ (B) CF_3_S-Pro *via* Stereospecific Dehydroxytrifluoromethylthiolation;^56^ (C) α-SCF_3_-β^2,2^-AAs *via* Asymmetric Catalysis;^57,58^ (D) CF_3_S-Leucine^59,60^

TFTI in combination with *N*-heterocyclic carbenes has also been used to form an intermediate CF_3_S-benzimidazolium salt, providing stereospecific dehydroxytrifluoromethylthiolation of alcohols.^56^ This method, compatible with various structurally complex alcohols, provided access to the optically pure (2*S*,4*S*)-4-SCF_3_ proline analog from commercially available 4-hydroxyproline in 76% yield (Scheme 10B). Two groups independently reported the synthesis of α-trifluoromethylthio-β^2,2^-amino acids by asymmetric trifluoromethylthiolation of α-substituted isoxazolidin-5-ones *via* chiral quarternary ammonium salt phase-transfer organocatalysis, followed by reductive N–O bond cleavage of the enantioenriched masked α-SCF_3_-β^2,2^-AAs (Scheme 10C).^57,58^ Waser, Cahard and co-workers further incorporated synthesized CF_3_S-β-AA into a model dipeptide and performed the CF_3_S post-oxidation to the corresponding sulfone derivative.^58^ Additional examples of CF_3_S-AAs have been reported as isolated examples without further application in peptide chemistry. Tertiary CF_3_S-leucine analogs were obtained either by AgSCF_3_/Na_2_S_2_O_8_-mediated oxidative trifluoromethylthiolation of inactivated aliphatic C–H bonds^59^ or by photoredox-mediated HAT using the phthalimide *N*-SCF_3_ reagent (Scheme 10D).^60^ In addition, a CF_3_S-substituted biaryl phenylalanine derivative was synthesized *via* Suzuki–Miyaura cross-coupling using the CF_3_S-arylboronic ester as part of a broader mechanistic investigation.^61^

### Hydrophobic Contribution Scale of Trifluoromethylthio-Containing Amino Acids in Peptides

In our research on the rational design of peptides featuring fluorinated amino acids, we investigated their hydrophobic contribution compared to canonical amino acids. For this purpose, we established a comparative and accurate scale of intrinsic hydrophobicity of amino acids.^46^ Chromatographic hydrophobicity indexes (HI) were determined for model tripeptides containing varied residues at the internal position, enabling assessment of the local hydrophobicity contribution of each amino acid within a peptide (Figure 2).^46,48^

**Figure 2:**
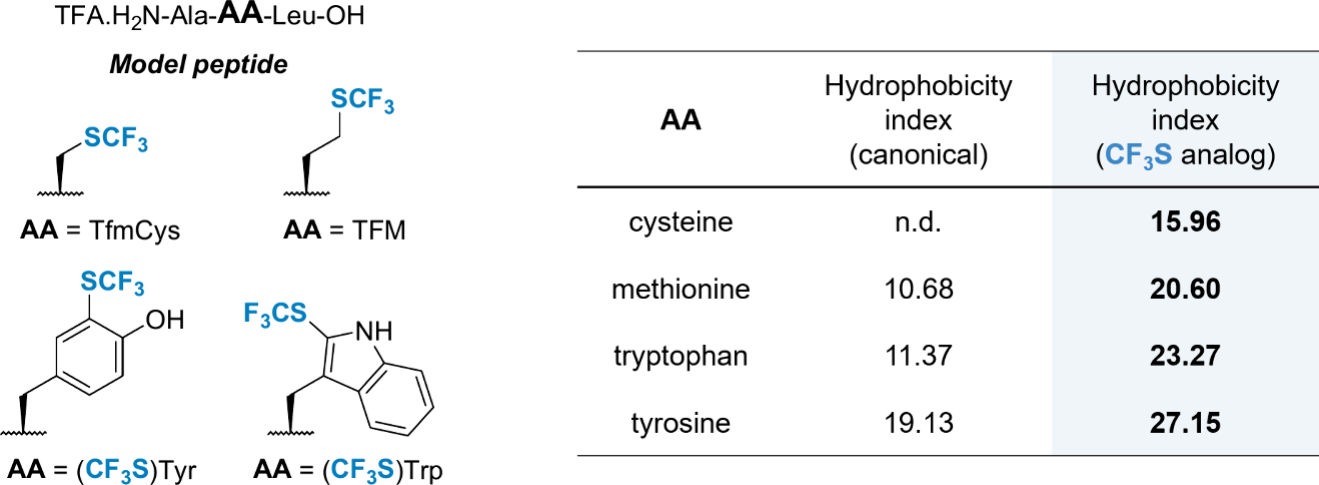
Hydrophobicity index (HI) scale of model tripeptides TFA·H_2_N-Ala-**AA**-Leu-OH comparing values for canonical and trifluoromethylthiolated amino acids at pH 7.^46,48^

Trifluoromethylthio-containing AAs showed significantly increased local hydrophobicity relative to their proteinogenic counterparts, with the magnitude depending on the nature of the respective side-chain. Trifluoromethylcysteine and trifluoro­methionine are significantly more hydrophobic than methionine or other aliphatic AAs.^46^ Aromatic CF_3_S analogs of tyrosine and tryptophan were the most hydrophobic AAs reported to date.^48^ Beyond hydrophobicity, side-chain functional group acidity and hydrogen bonding propensity are important for peptide–protein recognition and peptide folding. We observed that trifluoromethylthiolation of the Tyr residue increased the acidity of the phenolic hydroxyl ∼50-fold (p*K*_a_(H_2_O) = 8.1).^48^ This p*K*_a_ value is comparable to that reported for *ortho*-trifluoromethylated phenols.

## 3 CF_3_S Motif: A Sensitive Probe for Chemical Biology

### CF_3_S as a Label in ^19^F NMR Spectroscopy

The CF_3_S group, containing three magnetically equivalent fluorine atoms in an isolated spin system, can serve as a ^19^F NMR spectroscopy sensor for probing macromolecular structures and dynamics, as well as for monitoring RNA or protein binding events and ligand interactions at micromolar concentrations.^5^ However, its distinct stereoelectronic properties may perturb native structure and function, which should be considered when using it as a ^19^F NMR probe.

In 2012, Micura and co-workers reported the first trifluoromethylthio-labeled RNA, achieved by solid-phase incorporation of the 2′-SCF_3_ uridine unit (Scheme 11A).^11^ The ribose 2′-deoxy-2′-trifluoromethylthio building block was prepared in high yield on a multi-gram scale *via* *S*-trifluoromethylation using the Togni reagent.^11^ 2-SCF_3_ uridine exhibited high stability when subjected to coupling, deprotection, and oxidative conditions of standard solid-phase RNA synthesis. While the label did not alter the secondary structure of single-stranded regions, it disrupted planar base-pairing and thermodynamically destabilized RNA duplexes.^11^ This effect was attributed to the modified nucleoside’s preference for the C2′-*endo* ribose conformation, rather than the native C3′-*endo* conformation characteristic of A-form RNA duplexes, leading to steric interference and weakened stacking and H-bonding interactions.^12^ Compared to 2′-SCH_3_ uridine modifications, which only slightly destabilize duplexes, the diminished electronegativity of sulfur in SCF_3_ plays a significant role. In a subsequent study, the authors expanded the repertoire of 2′-SCF_3_ nucleosides with adenosine and guanosine phosphoramidite analogs suitable for solid-phase RNA synthesis.^13^

**Scheme 11:**
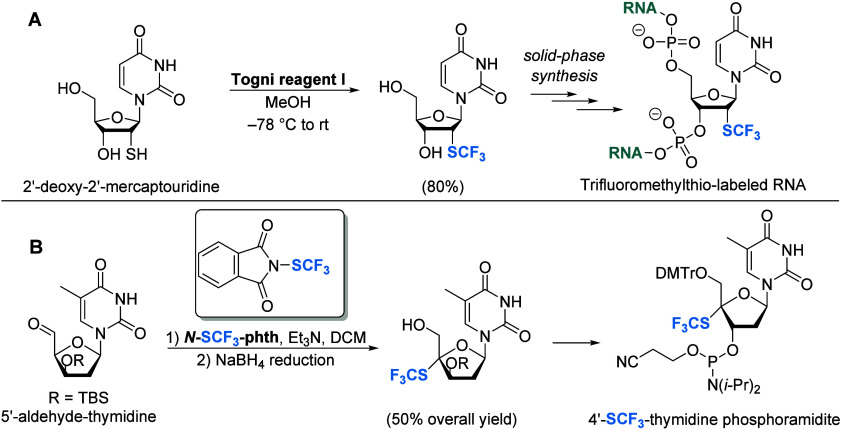
(A) Synthesis of 2′-SCF_3_-Uridine-Labeled RNA;^11^ (B) Synthesis of 4′-SCF_3_-Thymidine Phosphoramidite *via* Electrophilic Trifluoromethylthiolation^14^

Plavec, Zhou and co-workers reported the synthesis of 4′-SCF_3_-thymidine phosphoramidite and its incorporation into DNA using standard solid-phase protocols (Scheme 11B).^14^ Protected 5′-aldehyde-thymidine underwent stereoselective electrophilic trifluoromethylthiolation with an *N*-SCF_3_ phthalimide reagent, followed by NaBH_4_ reduction to yield 4′-SCF_3_-deoxythymidine, which was converted to the corresponding phosphoramidite for automated DNA synthesis.^14^ In contrast to the structural perturbation caused by the 2′-SCF_3_ modification in RNA, structural analysis showed that the 4′-SCF_3_ substitution only slightly constrained the deoxyribose conformation and was well accommodated in DNA duplexes. ^19^F NMR studies demonstrated that the 4′-SCF_3_ label enables detection of single-nucleotide polymorphisms, DNA topology, and DNA–protein interactions at sub-millimolar concentrations. The authors recently extended the synthetic strategy to prepare 4′-SCF_3_-uridine phosphoramidite and incorporated it into RNA strands *via* solid-phase synthesis.^15^ The 4′-SCF_3_ modification was well accommodated within the minor groove and did not induce significant distortion of the RNA duplex. A specific interaction between the 4′-SCF_3_ and 2′-OH of the 5′-adjacent ribonucleotide was identified to be a key feature for RNA secondary structure elucidation with excellent sensitivity, both *in vitro* and in living cells.

In proteins, trifluoromethionine (TFM) has primarily been used for ^19^F NMR studies.^5^ In 1997, Honek and co-workers reported the first TFM bioincorporation into bacteriophage λ lysozyme, which contains three Met residues in its wild-type sequence.^7^ The native aminoacyl-tRNA synthetase recognized l-TFM, enabling overexpression of LaL with high (70%) or partial (31%) methionine substitution. The labeled lysozyme retained wild-type folding and catalytic activity, and exhibited increased hydrophobicity. In a later study, Holzberger et al. reported 82% l-TFM substitution of 14 Met residues in DNA polymerase I (*KlenTaq*, 63 kDa).^9^ Despite most Met sites being located in the hydrophobic core of the polymerase, the TFM-modified enzyme retained high activity and substrate selectivity, albeit with moderately reduced stability. The CF_3_S label was used to investigate the dynamics of substrate recognition and DNA synthesis. Internalized TFM residues exhibited broader ^19^F resonances than flexible surface-exposed sites, but conformational changes of the enzyme during nucleotide incorporation could be clearly detected. TFM labeling has also been applied to investigate larger assemblies and synthetic peptides. In 2017, Davis and co-workers used TFM to monitor the disassembly of virus-like particles (VLPs) by ^19^F NMR.^10^ Analysis of CF_3_S resonances allowed assessment of VLPs assembly state and degradation level when exposed to denaturants or reductants, which guided particle design for controlled VLP-carried cargo release in cells. In peptides, site-selective incorporation of TFM into the amyloidogenic Aβ_1–40_ peptide *via* Fmoc-SPPS enabled real-time monitoring of oligomer formation and irreversible aggregation by ^19^F NMR.^6^ Other TFM derivatives, particularly difluoromethionine (DFM) and 2,2,2-trifluoroethanethiol cysteine (TFET-Cys), have also been used as ^19^F NMR probes.^5,62^ As the repertoire of SPPS-compatible CF_3_S-AAs continues to expand, new opportunities are emerging for ^19^F-labeled peptides to investigate structural transitions and dynamics.

### [^18^F]­SCF_3_ Construction for Applications in ^18^F-Based PET Imaging

In positron emission tomography (PET) imaging, incorporation of the ^18^F isotope into small molecules or peptides is one of the key strategies for the preparation of radiotracers.^63^ Several methods for synthesizing ^18^F-labeled trifluoromethyl sulfides have emerged in the past decade and have been partially reviewed elsewhere.^63,64^ In 2015, Gouverneur and co-workers reported the first construction of [^18^F]­SCF_3_ moiety *via* Ag­(I)-promoted halogen exchange between C­(sp^2^)–SCF_2_Br precursors and [^18^F]­KF/K_222_, affording unprecedented [^18^F]­CF_3_S-functionalized compounds in 12–92% radiochemical yield (RCY) (Scheme 12A).^65^

**Scheme 12:**
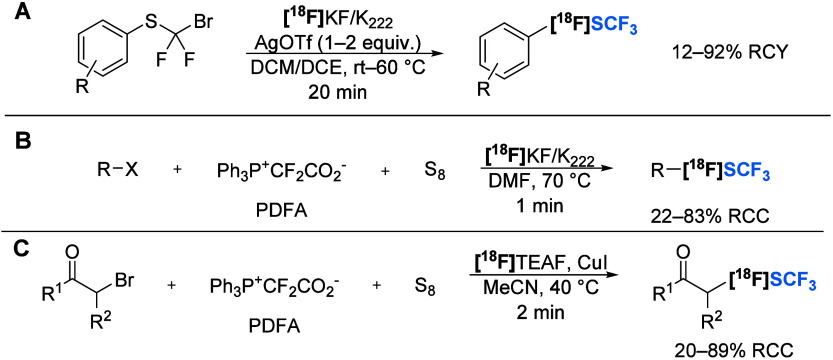
Seminal Synthetic Methods for the Preparation of [^18^F]­CF_3_S-Containing Compounds

Concurrently, Zheng and co-workers developed a transition-metal-free exogenous fluoride-mediated method for [^18^F]­CF_3_S-radiolabeling of (hetero)­benzyl and alkyl halides based on difluorocarbene chemistry (Scheme 12B).^66^ *In situ* decarboxylation of difluoromethylene phosphobetaine (PDFA) generates difluorocarbene, which is readily trapped by radiolabeled fluoride and further converted to the [^18^F]­CF_3_S^–^ anion in the presence of elemental sulfur. This anion undergoes nucleophilic substitution at benzyl or alkyl position in moderate to high radiochemical conversion (RCC).^66^ The method tolerates diverse functional groups, and is compatible with ^18^F-labeling due to its sub-1 min reaction time. An extension of this approach employed Cu­(I)-catalyzed [^18^F]­trifluoro­methyl­thiol­ation of α-bromo carbonyl derivatives using *in situ*-generated ^18^F-labeled tetraethylammonium fluoride (TEAF), affording α-[^18^F]­CF_3_S carbonyl derivatives in moderate RCC (Scheme 12C).^67^ Subsequent advances of these seminal methods include the use of radiolabeled [^18^F]­fluoroform for rapid conversion of diaryl disulfides to [^18^F]­CF_3_S-substituted arenes in moderate radiochemical yield (45–74% RCY, >95% purity).^67^ Gouverneur, Shen and co-workers further expanded the precursor scope to BrCF_2_S-substituted arenes by utilizing corresponding (hetero)­aryl boronic pinacol esters, which underwent Ag-mediated halogen exchange ^18^F-labeling to yield novel [^18^F]­CF_3_S (hetero)­arenes.^68^ This strategy also enabled preparation of [^18^F]-Umemoto reagent, an electrophilic [^18^F]­CF_3_ donor for site-selective radiolabeling of cysteine residues in peptides (Scheme 13A).^69^ Optimized reaction conditions provided high chemoselectivity for Cys residues in the presence of most proteinogenic AAs (except Trp and His), affording biologically relevant [^18^F]­CF_3_S-labeled peptide analogs such as glutathione, a prostate-specific membrane antigen (PSMA) radioligand, a β-amyloid fragment, and cyclic RGD in high RCY (10–33%). *In vivo* study of the RGD conjugate confirmed the expected biodistribution and stability with no ^18^F-fluoride elimination detected. l- and d-[^18^F]­TfmCys were also evaluated as PET tracers for glioma imaging.^70^

**Scheme 13:**
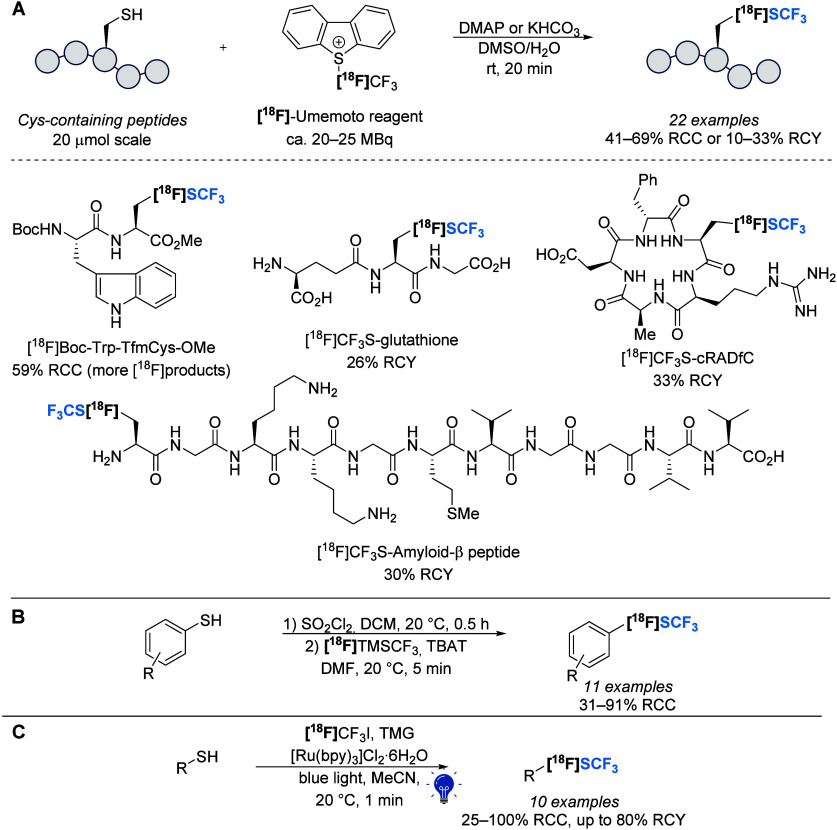
(A) ^18^F-Labeled TfmCys-Containing Biologically Relevant Peptides;^69^ (B) Synthesis of [^18^F]­SCF_3_-Labeled Arenes from Corresponding Thiophenols Using the [^18^F]-Labeled Ruppert–Prakash Reagent;^71^ (C) Synthesis of ^18^F-Labeled Trifluoromethyl Sulfides Using [^18^F]­Trifluoroiodomethane^72^

More recently, ^18^F-labeled CF_3_S­(O)_*n*_ (*n* = 0, 1, 2) substituted arenes were prepared from thiophenols or aryl sulfonyl fluorides using the [^18^F]-labeled Ruppert–Prakash reagent (Scheme 13B).^71^ A one-pot process proceeding *via* sulfenyl chloride intermediate gave [^18^F]­CF_3_S products in 31–91% RCC with high functional group and heterocyclic substrate tolerance. Related ^18^F-labeled sulfoxides and sulfones, including PET tracers such as clofibrate and celecoxib were also prepared on a larger scale. Moreover, a recent study reported a photoredox-mediated synthesis of ^18^F-labeled trifluoromethyl sulfides using [^18^F]­trifluoroiodomethane, achieving moderate to high RCC (Scheme 13C).^72^ The development of [^18^F]­CF_3_I represents an important milestone in radical, photoredox-mediated [^18^F]­trifluoromethylation chemistry and offers new opportunities in ^18^F-labeling.

## 4 Conclusion and Future Perspectives

This Synopsis highlights the unique effects of CF_3_S-substitution, recent methodological advances enabling its incorporation into biomolecules, and its potential as a ^19^F NMR and ^18^F PET label. Among the steadily expanding repertoire of synthetic methods for the CF_3_S-group introduction, recent studies on trifluoromethylthiolation of aromatic AAs and its resulting impact on peptide properties are particularly noteworthy. Site-selective incorporation of the trifluoromethylthio group into biomolecules has been shown to address inherent pharmacokinetic limitations of peptides by increasing their metabolic stability and bioavailability through enhanced hydrophobicity. Interestingly, the stereo-electronic features of the CF_3_S group do not present major drawbacks regarding the selectivity of the interactions of the CF_3_S-analogs with their biological targets. Robust preparation of diverse enantiopure CF_3_S-AA building blocks on a multi-gram scale and their efficient implementation in SPPS now enable specific application in yet unexplored systems such as membrane-active or macrocyclic peptides, among others. For the future perspectives, development of biocompatible and late-stage site-specific modification method for incorporation of the CF_3_S group into complex biomolecules such as native peptides and proteins should also be considered to avoid *de novo* synthesis. The physicochemical and biological activity studies conducted by our group and others contribute to a more detailed understanding of the impact of sulfur-associated fluorinated motifs on biomolecule properties. These developments provide a basis for further evaluation of emergent chalcogen-associated fluorinated groups such as (a) CF_3_S­(O)_*n*_ (*n* = 1, 2); (b) CHF_2_S, CH_2_FS, CF_3_CF_2_S; (c) CF_3_S–N;^73^ (d) CF_3_Se­(O)_*n*_ (*n* = 0, 1, 2) and sulfur–selenium-associated motifs^74^ in the rational design of small molecules, peptides, proteins, and nucleic acids with tunable stability, bioactivity, and imaging capabilities.

## Data Availability

The data underlying this study are available in the published article.
